# A Review on the Use of Impedimetric Sensors for the Inspection of Food Quality

**DOI:** 10.3390/ijerph17145220

**Published:** 2020-07-20

**Authors:** Shan He, Yang Yuan, Anindya Nag, Shilun Feng, Nasrin Afsarimanesh, Tao Han, Subhas Chandra Mukhopadhyay, Dominic Rowan Organ

**Affiliations:** 1 School of Chemistry and Chemical Engineering, Guangzhou University, Guangzhou 510006, China; he0091@gmail.com (S.H.); marktoon1023@163.com (Y.Y.); 2 Flinders Institute of Nanoscale Science and Technology, College of Science and Engineering, Flinders University, Bedford Park, South Australia 5042, Australia; 3 DGUT-CNAM Institute, Dongguan University of Technology, Dongguan 523000, China; afsarimanesh.n@gmail.com (N.A.); hant@dgut.edu.cn (T.H.); 4 School of Electrical and Electronic Engineering, Nanyang Technological University, Singapore 639798, Singapore; 5 School of Engineering, Macquarie University, Sydney 2109, Australia; subhas.mukhopadhyay@mq.edu.au; 6 Department of Social Sciences, Heriot-Watt University, Edinburgh SC000278, UK; dominic.organ@gmail.com

**Keywords:** sensors, impedimetric, food, quality, biomolecule

## Abstract

This paper exhibits a thorough review of the use of impedimetric sensors for the analysis of food quality. It helps to understand the contribution of some of the major types of impedimetric sensors that are used for this application. The deployment of impedimetric sensing prototypes has been advantageous due to their wide linear range of responses, detection of the target analyte at low concentrations, good stability, high accuracy and high reproducibility in the results. The choice of these sensors was classified on the basis of structure and the conductive material used to develop them. The first category included the use of nanomaterials such as graphene and metallic nanowires used to form the sensing devices. Different forms of graphene nanoparticles, such as nano-hybrids, nanosheets, and nano-powders, have been largely used to sense biomolecules in the micro-molar range. The use of conductive materials such as gold, copper, tungsten and tin to develop nanowire-based prototypes for the inspection of food quality has also been shown. The second category was based on conventional electromechanical circuits such as electronic noses and other smart systems. Within this sector, the standardized systems, such as electronic noses, and LC circuit -based systems have been explained. Finally, some of the challenges posed by the existing sensors have been listed out, along with an estimate of the increase in the number of sensors employed to assess food quality.

## 1. Introduction

The intervention of sensors in the application world has helped in developing the quality of life of human beings on a daily basis, starting with the popularization of semiconducting sensors, around three decades ago [1,2,3], which have been implemented primarily for industrial and environmental applications. Initially, the prototypes based on silicon substrates were used [4,5,6] for sensing purposes. These are non-flexible or rigid sensors that are formed using conventional Microelectromechanical Systems (MEMS)-based techniques. Non-flexible materials with moderate or high brittle nature are used as raw materials to develop these sensors. These materials are processed using lithographic techniques, where the structure and dimensions have been considerably reduced to form microelectronics. The non-flexile sensors have a high versatility to be implemented for certain industrial [7,8,9] and environmental [10,11,12] applications. They have also been used in other forms, where a particular quality of this material had been used for quantum mechanical studies. For example, silicon nitrate solutions have been prepared to determine the strong quantum squeezing for the detection of low-count photons [13] and scan probe microscopies [14]. Silicon has been typically used to form silicon nitrate solutions having Young’s modulus, Poisson’s ratio, and material density of 2.5 × 10^11^ Pa, 0.23, and 3.1 × 10^3^ kg/m^3^, respectively. These solutions were subsequently used as thin films to coat metals and probes for detecting photons. 

Although these sensors are very popular, there are some limitations associated with them, which leads to subsequent limitations to their applications. Some of those limitations are high input power, degradation in their sensitivity with time, highly brittle nature, relatively shorter life cycle, and the generation of toxic products during its fabrication [15,16]. This led scientists to opt for alternative options where the raw materials are flexible in nature. The flexible materials have been processed to develop sensors that had enhanced electrical, mechanical and thermal characteristics. Unlike silicon-based sensors, these sensors have the capability to be bent to a certain degree, depending upon the mechanical attributes of the electrodes and substrates of the prototypes. A wide range of techniques, including soft-lithography and printing processes, have been used to fabricate these sensors. A comparison between the pros and cons of the flexible and non-flexible sensors is given in Table 1. It is seen from the table that both types of sensors have certain advantages and disadvantages associated with them. So, a review of the exploitation of both these types of sensors for determining the food quality has been presented in the paper. 

In terms of sensing principles, some of the primary ones which have been focused upon by the researchers are impedimetric [17] and electrical [18] sensing. Most of the standardized sensors working in the microelectronics industry operate on either of these sensing mechanisms. The choice of a particular working principle of a prototype mainly depends on the application of the sensor. For example, when the sensors are used to detect very low applied force [19], the changes in the capacitive values would be determined. Among these options, impedimetric sensing has been largely preferred [20], as it helps to obtain a large amount of information related to the target analyte. This type of sensing has certain pros and cons related to it. 

Although there are some disadvantages, such as short shelf life, a high probability of cross-sensitivity and high sensitivity towards the change in ambient temperature, they offer a lot more advantages in terms of static sensor characteristics. Some of them are low detection limit (LOD), good stability and reproducibility in the responses, the ability to respond to a wide linear range, low power requirement, portability good resolution, high repeatability and high accuracy [21]. Among the popular usages of these sensors for industrial applications, the detection in the food quality holds the pivotal spot in the current era. In today’s world, more advanced food-producing techniques have been developed in comparison to earlier times. Today, in the era of consumerism, food products are developed on a very large scale, and in a very quick time. This makes it necessary to avoid adulteration of the products anyway. Moreover, the high demand for a different kind of food material brings about a plausible option for them to be stored in the local stores and supermarkets in a large quantity and for a long time. The storage of food and beverages for a long time also, however, deteriorates their quality, which, if consumed even in a small amount, may drastically affect the health of a person [22,23]. 

**Figure 1:**
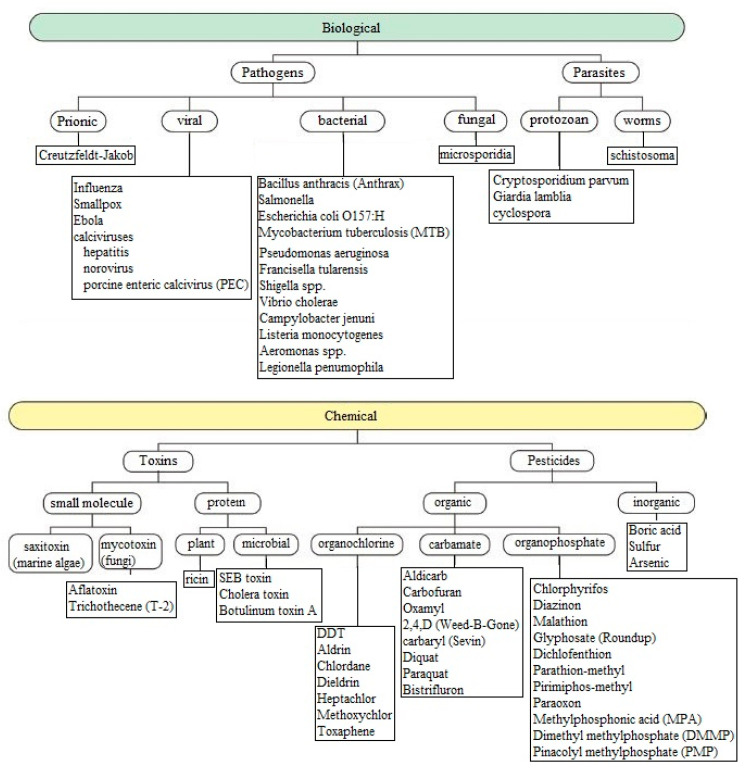
Representation of a hierarchical categorization of the analytes that are present in food and water [24].

The use of sensors in this area is currently one of the practical ways of achieving delicate balance due to its capability to operate in harsh conditions, perform chemical fingerprinting of the specified species, ability to deal with the biomolecules present in the food matrix, and a wider range of detection. Farahi et al. [24] had very neatly described the critical issues related to food and water safety that are being addressed and resolved by these sensors. Advanced receptor-based and non-receptor-based sensors are being engineered to obtain prototypes with better performance in terms of sensitivity and efficiency. Some of the pathogens that are being detected in food and water are shown in Figure 1 [24]. These pathogens are being dealt with integrated sensing systems that operate on lap-on-a-chip systems, where the sensors are based on biological and chemical-based receptors. For example, certain bio-catalysis models such as the “lock-and-key model” have been largely preferred [25,26] for the selective detection of biomolecules present in food, due to certain attributes, such as its simplicity and high specificity on the enzyme. 

For the food products developed at home, ubiquitous sensing prototypes need to be developed, which can be employed to test the condition of the material on a regular basis. The quality of the food products processed on an industrial scale is highly vulnerable, as an aberration, even on a very small scale, can create health-related problems on a large scale. On the large-scale production of food in the industries, sensors are classified on the basis of the nature of the product [27,28]. Each of the different sensors has specific nutrient content associated with it. The measurement of this content is extremely important to determine the overall amount of intake in the body of a person. The anomaly caused due to prolonged exposure to this content may create a permanent misbalance, thus affecting the health of the person. 

Researchers have been trying to develop sensors [29,30,31,32,33] that can determine the exact composition of the food and beverages produced in the industries. The sensors have been developed to detect the complex matrix of food samples in order to trace micro-scaled amounts of hazardous agents. These sensors have been fabricated to achieve a high-throughput with low-cost fabrication, accuracy, and high sensitivity for food testing [34,35]. The prototypes have been developed using singular as well as a combination of materials, depending upon their characteristics. In some cases, the fusion of two or more materials helps to increase the sensitivity and selectivity of the prototypes. This is because, with the formation of nanocomposites, the individual properties of the conductive fillers and polymeric matrix get amalgamated for further enhancement of the generated compound. For example, due to the exceptional properties of graphene, its mixing in a polymer matrix results in nanocomposites showing a substantial enhancement in their multifunctional aspects [36]. The output material becomes stronger with simple processing. 

In some cases, metallic nanoparticles are also instructed alongside graphene to facilitate an improvement in the inter-particle contact in the host matrix. This also helps in increasing the homogeneity in the developed nanocomposites. Like the utilization of ceramic reinforced graphene composites has been largely done in the chemical and biological fields due to their high stability, robustness, inertness towards other chemicals and recyclable nature. The range of polymeric materials that have been mixed with the nanofillers also varies depending upon their requirement. One of the cases can be seen in [37], where a conductive polymer such as poly (3,4-ethylenedioxythiophene) polystyrene sulfonate is mixed with palladium and graphene to generate highly sensitive, wearable thin-film strain sensors. One of the common raw materials for sensors that have been popularized for food-testing is carbon allotropes, due to their simple structure, high portability, and the ability to deal with certain disadvantages, such as a lack of robustness, low sensitivity, and an incapability to sense the quality of multiple biomolecules present in the food products [38,39,40,41,42,43,44,45]. Different kinds of carbon materials, such as Carbon Nanotubes (CNTs), graphene, and graphene oxide, have been employed [46,47] to form the sensors for food testing, but none of the review papers have explicitly categorized the sensors for food-testing based on their operating principle and processed material. This paper showcases the use of some of the sensors based on their physio-chemical characteristics, such as size, dimensions and their capability to induce high selectivity and sensitivity towards the chosen molecule. 

The range of impedimetric sensors has been categorized on the basis of the raw materials used to develop them. Table 2 showcases the summary of a few of the selected studies done with the use of carbon-based nanomaterials on the determination of food quality, based on essential parameters. It can be seen from the table that even though each of these prototypes has been formed using different materials, the choice of the processed material has been specifically based on its high selectivity. Even though some of these works have achieved high sensitivity with the fabricated prototypes, all these sensors have been labeled for increasing their specificity towards the molecule under consideration. For example, the detection sensitivity of the graphene oxide-based sensors detecting kanamycin [48] was as low as 100 fM, but these sensors were labeled with Flourescein amidite (FAM)- labelled kanamycin-binding aptamer (KBA) to increase their detection capability towards a series of tested kanamycin concentrations ranging between 1 pm and 1 µM. It is a time-consuming process as well as it increases complications in the system. Moreover, the labeling of the sensors fixes the concentrations of the tested kanamycin samples. 

With label-free graphene-oxide sensors, the approach would be much simpler, and it would obtain more direct information with the hitting and validating technique [58]. On the contrary to the detection of kanamycin, a very high sensitivity of 260.75 mA/mM was obtained for the detection of glucose [54] with the label-free field-effect transistors. These devices have been able to detect glucose at very low concentrations of 0 to 30 mM. These sensors should be encouraged for rapid detection and high stability. 

The sections of the paper are organized in the following manner. Section one highlights the necessity of the detection of food quality, along with the significance of sensors for this application. Section two mentions the different types of impedimetric sensors that have been fabricated and utilized for the inspection of food quality. This section has been sub-categorized based on the conductive materials that are used to form the sensors. The two sub-classes that are predominantly used for detection of food quality are nanomaterial-based sensors and conventional electronic sensors. Among the first category, explanations related to different forms of graphene and metallic nanoparticle-based sensors have been done. For conventional sensors, electronic nose and other embedded circuitries have been elucidated. Section three shows some of the challenges currently faced by the existing sensors in this application, in addition to some of the novel ideas that can be implemented in this application. Finally, the conclusion is given in the last section of the paper.

## 2. Type of Impedimetric Sensors Utilized for the Inspection of Food Quality

Among the sensors that have been developed and implemented to determine the quality of food, some of the major research work has been mentioned here [59]. MEMS and nanoelectromechanical (NEMS) and other printing techniques have been used to fabricate the sensors. In terms of raw materials, crystalline and amorphous elements have been used to form the electrodes and substrates of the sensors. Certain elements such as graphene nanosheets and metallic nanowires have also been chosen [60] to form the conductive part due to their excellent electrical, mechanical, and thermal properties. Various synthetic polymers have also been used to counteract some of the disadvantages of the MEMS- and NEMS-based sensors, such as the high cost of fabrication due to the requirement of cleanroom facilities, time-consuming processes, and the use of hazardous chemicals. These polymers also add certain advantages, such as stretchability, transparency, and flexibility [61,62]. These polymers include three types, namely elastomers, thermosets, and thermoplastics. Each of these categories has its own characteristics, the uses of which are based on the type of sensors that are developed for the detection of food quality. Some of the synthetic polymers that come under these categories are polydimethylsiloxane (PDMS), polyimide (PI), epoxy-based SU-8, polypropylene (PP), polystyrene (PS), poly (methyl methacrylate) (PMMA), polyethylene terephthalate (PET) and cyclic olefin copolymers (COC). Among them, the elastomeric polymers are the ones that serve the best for the fabrication of wearable devices, whereas the thermoplastics are favorable for the fabrication of disposable sensing devices.

The third category of substrates covers the ones developed from cellulose, which are inexpensive, porous, hydrophilic, transparent and have a biodegradable nature. The sensors formed with these substrates are highly applicable for point-of-care devices [63,64], due to their specific biodegradable coatings [65]. Cellophane-based substrates are other materials that are sub-categorized under this sector. They are mainly developed from wood, cotton, and other sources of cellulose. They are readily used for food packaging industries, and as disposable sensors [66,67]. The final category is called hybrid material, which combines two or more of the three types mentioned above. As shown in Table 2, different carbon allotrope-based materials have been preferred a lot as conducting material to form the sensors. Apart from carbon material, the deployment of electronic noses is a very standardized technique for food quality inspection, which consists of an array of sensors. The type of sensors may include metal-oxide-semiconductor field-effect transistor (MOSFET), metal oxides (MOX), or other commercial sensors, depending on the specific operation of the nose. 

The change in the quality of food is dependent on the growth of different kinds of microorganisms, which makes the food inedible. The sealed and loose packets containing food may be a site for bacterial growth, as a result of leakage and exposure to moisture. The resulting decrease in food quality creates problems for consumption, as well as a commercial loss because of the perishing of the product. The shelf life of food products constantly changes depending upon the senescence, microbial spoilage, and chemical deterioration, which ultimately leads to rancidity and growth of pathogens [68]. Some of the techniques to preserve the quality of food include storage at a low temperature, controlled atmospheric packaging, pasteurizing, and deliberately increasing the acidity content of the food. The requirement of constant monitoring of the food quality makes it necessary to develop integrated sensing prototypes. 

### 2.1. Impedimetric Sensors Based on Graphene and Other Nanomaterials

The use of impedimetric sensors to determine the quality of food and beverages has been a very popular research topic in recent years [69]. Among them, nanomaterials have been largely preferred due to their enhanced electromechanical characteristics, high surface-to-volume ratio, and high specificity. The utilization of a lot of nanomaterials, including graphene and other forms, has been explained in the next two sub-sections. These materials have used to form impedimetric sensors for rapid, efficient and economical sensing [70,71]. Various type of nanomaterials has been considered [72,73] to form sensors with high sensitivity and selectivity. Some of the common ones are graphene [74,75], gold [76,77], and mono- and bimetallic nanoparticles and metallic nanocomposites [78,79]. Researchers have been working on designing and fabricating biosensors with resources having enhanced electromechanical and thermal characteristics. For example, Avramescu et al. [80] have depicted the use of screen-printed graphite-based sensors operating as amperometric detectors. The sensors have been used as disposable prototypes formed with NAD+-dependent dehydrogenases, tyrosinase, and genetically modified acetylcholinesterases. Some of the elements that have been detected using these sensors are D-lactic acid, L-lactic food, and acetaldehyde. Real-time samples, such as wine, were tested for validating the performances of these prototypes. Below shown are some of the examples related to the use of graphene and other nanomaterials for the detection of food quality. Each of the devices used the conductive material in a certain form that would assist them to achieve the highest selectivity and sensitivity towards the target molecule. 

#### 2.1.1. Graphene-Based Impedimetric Sensors

Graphene, due to its unique electrical properties, has largely been used for the quality assurance and safety of food [75,81]. Some of the attributes of graphene that have been essential for the detection of food quality are high adsorption capability due to high surface area, high electrical conductivity, and high catalytic activity [82]. The use of graphene to form nano-hybrids has also been shown by Chen et al. [83], where the primary aim was to detect the presence of oxalic acid (OA) in human bodies. Since the excess amount of OA, as obtained from consumed fruits and vegetables, creates a complex with the available calcium ions in the body, it is required to control their concentration to avoid certain organs like stomach and kidneys from getting affected. The graphene nanosheets were mixed with platinum nanoparticles to form the hybrid for impedimetric detection of OA. The sensing phenomena were based on cyclic voltammetry (CV) and differential pulse voltammetry (DPV) techniques. The electrochemical oxidation of oxalic acid took place on the platinum nanoparticle-loaded graphene nanosheet-modified electrodes, as a result of which, well-defined peaks were obtained. A notable decrease in the overpotential was also obtained, in comparison to those of the bare or graphene nanosheet-modified electrode. The range and LOD of OA were from 0.1 to 50 mM, and 0.1 mM, respectively. 

Similar to platinum nanoparticles, palladium was also used alongside graphene to form conductive hybrids for sensing purposes [84]. This hybrid was placed on film-coated glassy carbon electrodes for the detection of hydrogen peroxide, present in foods, pharmaceutical, industrial, and environmental analysis. Voltammetric and amperometric techniques were employed to conduct the impedimetric experiments with the palladium nanoparticle-loaded graphene nanosheet-modified electrodes. The modified glassy electrodes demonstrated direct and mediatorless responses to the target analyte at low potentials. The LOD for these experiments was 0.05 µM, whereas the measured range lay between 0.1 and 1000 µM. Some of the colorants, such as sunset yellow (SY) and tartrazine (TT), present in aromatic dyes, are very harmful to human beings if consumed in high amounts. These colorants are responsible for causing certain anomalies, such as migraines, allergies, anxiety, diarrhea, and even cancer.

Another work where graphene-based materials have been used for quality testing was shown by Gan et al. [85]. They proposed a novel idea where the graphene layer was wrapped using phosphotungstic acid (PTA) to form selective material for detection purposes. Impedimetric responses were determined based on the graphene-based electrodes. Th electrocatalytic activities of the prototypes were studied where the oxidation of the target samples led to the enhancement of the significant peak current and the lowering of the oxidation overpotential. Further optimization on other experimental parameters, such as supporting electrolyte, the volume of the electrolyte suspending the electrodes, accumulation potential and time, assisted in decreasing the LOD and increasing the peak current. These devices had a high sensitivity for the detection of SY and TT, obtained a peak potential of around 260 mV with the pulse voltammetry technique. The LODs obtained for the measurements of SY and TT were 30 and 0.5 µg/L, respectively. This hybrid of graphene and PTA was also mixed with glassy carbon electrodes [86,87] for detecting other colorants, such as Sudan I and Orange II. The detection range was from 3.3 to 660 nM, and from 2.85 to 28.54 nM for Sudan I and Orange II, respectively. Similar to nanoparticles, certain compounds like tin dioxide could also be used with graphene to form nanocomposites for the detection of deadly contents like mercury in very low concentrations in food [88]. The prototypes showed high electro-catalytic activities, performing the reduction of mercury ions during the experimental process. 

Other forms of graphene-like quantum dots were mixed with certain nanoparticles like gold in glassy carbon electrodes to detect malachite green [89], having an LOD of 1 × 10^−7^ mol/L. The magnetic composites were also used by other researchers [90,91] for the extraction of certain analyte and triazine herbicides, such as atrazine, prometon, propazine, and prometryn. Some of the other works using graphene-based devices for the detection of pesticides are [92,93,94,95,96]. Apart from these biomolecules, other nanomaterials, such as metallic nanoparticles [97] and carbon nanomaterials [98], have also been used for the detection of harmful chemicals present in the food products. Another work depicting the use of graphene for inspecting the quality of food was shown by Migliorini et al. [99], where reduced graphene oxide was used for the detection of malathion pesticide. The devices were formed using polymeric nanofibers, having a combination of polyamide 6 and polypyrrole, that were electrospun together along with graphene. Impedimetric experiments were carried out using CV and electrochemical impedance spectroscopy (EIS) to determine the changes in the responses of the developed prototypes for the tested concentrations of malathion pesticide. For CV, the graphene-based electrode served as the working electrode, whereas platinum foil and Ag/AgCl/KCl were used as the counter and reference electrodes, respectively.

EIS measurements were performed with KCl containing ferrocyanide ions as redox probes with a potential open circuit. The sensing surface of the prototypes was modified using chemically and electrochemically reduced graphene oxides to increase the sensitivity and improve the LOD. The LOD and signal-to-noise ratio were 0.8 ng/mL and 3, respectively. The three-electrode system consisted of 0.1 M sodium sulfate solution with a voltage and scan rate of −1.5–1 V and 50 mV/s, respectively. For IS measurements, the frequency sweep was done between 0.1 and 100 kHz, having 0.1 mol/L KCl as a redox probe. The buffer solution had a pH of 7.4, having input parameters of voltage, pulse width and a pulse period of 50 mV, 0.4 s and 0.5 s, respectively. The fabricated prototypes were able to detect malathion in real samples in the presence of other pesticides such as pestanal and cadusafos by depicting a change in current. The reproducibility and recoveries of malathion from the real-time samples were 1.4–2% and 99–105%, respectively. 

Table 3 shows some of the other uses of graphene-based sensors for the detection of harmful biomolecules in food for assurance and safety. It is seen that the ligands vary from sensor to sensor, wherein nanoparticles, compounds and alloys have been used alongside graphene to form active sites for the electro-catalytic activity in the detection of graphene. Similar to Table 2, some of the research works [100,101] mentioned in Table 3 have used labeled sensors to perform the experiments. The labeling of these graphene sensors induces high complexity since some of the active sites generated through the developed immuno-complex does not respond to the intended analyte [102], which eventually decreases the overall sensitivity. Also, some of the disadvantages associated with the conjugates are the formation of aggregation and resistance to the used drugs [103]. In comparison to that, label-free sensors can achieve lower detection limits and wider linear ranges, as shown in [104]. The label-free sensors also increase the synergic effects of the sensors, which are blocked in the case of labeled sensors due to the secondary validation templates provided in them. 

**Figure 2:**
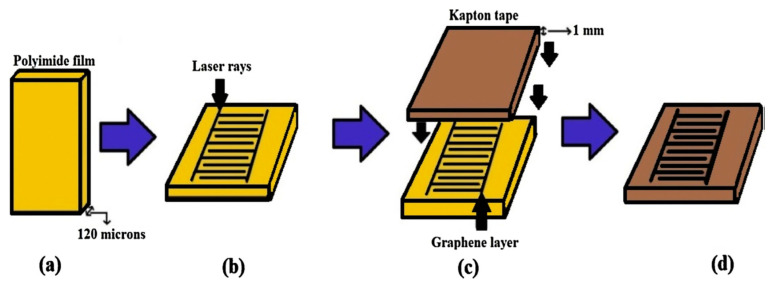
Schematic diagram of the steps of fabrication regarding the laser-induced graphene from polyimide films [27]. (**a**) The commercial polymers were (**b**) laser-induced to form graphene. (**c**) This graphene was transferred to Kapton tapes to form (**d**) the final prototypes.

One of the works, as done by Nag et al. [27], was related to the design, fabrication, and implementation of laser-induced graphene-based sensors for taste sensing purposes. The sensors were used to test the five fundamental tastes of sweet, sour, bitter, salty, and umami. The prototypes were formed by the laser-ablation of commercial polymer films. Figure 2 [27] shows the schematic diagram of the fabrication process of the sensor patches. Commercial polymer films were attached to glass substrates and taken for the laser ablation process. Certain laser parameters, such as power, speed, and *Z*-axis, were optimized prior to the laser induction. Laser-induced graphene was produced at the end of the ablation process. This graphene was generated due to the conversion of the sp^3^ hybridized carbon atoms in the polyimide films to the sp^2^ hybridized carbon atoms in graphene. The change in hybridization took place as a result of the breakage of bonds of carbon with other elements due to the local heat generated by the laser. After the generation of graphene, this conductive material was manually transferred to sticky tapes to use them as electrodes. Sticky Kapton tapes were placed carefully over the formed graphene, followed by manual transfer, to transfer them from the polyimide films to the tapes. The pressure was applied carefully to avoid damaging the design of the formed electrodes.

Interdigitated electrodes were formed that responded towards the tested samples through the change in their impedance values. The five different tasted samples were tested with concentrations ranging between 1 and 1000 ppm in order to determine the change in the resistance and reactance values of the sensors. The EIS technique was used to determine the responses of the sensors. An impedance analyzer was connected to the sensors via Kelvin probes to determine the changes in resistive and reactive parts of the impedance values. The impedance analyzer, in turn, was connected to the laptop using a USB to store the data using an automated data acquisition algorithm. The frequency sweep was done between 1 and 10 kHz. The sensors were capable of distinguishing each of the concentrations for the entire frequency sweep. These experiments were conducted for five chemicals, with each of them corresponding to a specific taste. The response time was two minutes, whereas the recovery time was ten minutes. A particular frequency showed that the reactance values were different for the five chemicals for all the tested concentrations. Other works on the use of laser-induced graphene for the detection of biomolecules in food are [116,117]. A summary of the above-mentioned research works on graphene-based sensors has been provided in Table 4. 

#### 2.1.2. Other Nanoparticle-Based Impedimetric Sensors

Apart from graphene and other carbon allotropes, there are other nanomaterials as well, which have been used to form biosensors for the inspection of food quality. These materials have been largely used for the chemical and microbiological analysis due to their quick response, high porosity, inexpensive for roll-to-roll production and great reliability [118,119]. These sensors have been able to operate on variant categories for food inspection, such as the detection of pathogens, toxins, adulterants, vitamins, pesticides, taste and smell. 

**Figure 3:**
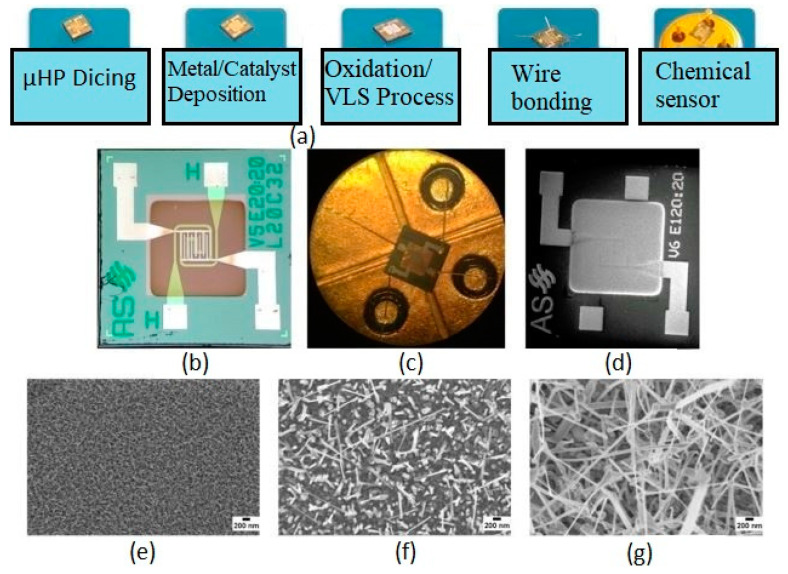
(**a**) Schematic diagram of the fabrication of the nanowires on the hotplates. (**b**,**c**) Optical images of the three types of nanowires. (**d**) Scanning Electronic Microscopic (SEM) image of the tin oxide-based sensor at low magnification. (**e**–**g**) SEM images of the tungsten trioxide, copper oxide and tin oxide nanowires, respectively [120].

Zappa et al. showed that [120] certain types of nanomaterials, such as tungsten, copper and tin, were being used to form oxide-based nanowires for the detection of food preservatives. These nanowires were subsequently used to form sensor arrays for low-power detection processes. These nanowires were deposited silicon micro hot plates using the MEMS technique. The sensor arrays were used for the discrimination of four types of compounds, namely ethanol, acetone, nitrogen dioxide and ozone. These compounds are said to be added to the food products to increase their shelf life. Each of the sensors in the developed arrays consumed power less than 50 mW and operate on the principle component analysis (PCA) technique. Figure 3a [120] shows the schematic diagram of the mounting of the nanowires on the hotplates to form the chemical sensors. The hotplates were initially diced and deposited with a metallic layer using the magnetron sputtering technique. The choice of the deposited metal depended on the nanowires as the former acted as a catalyst for the latter one. Then, the oxidation process was carried out to synthesize the nanowires directly on the hotplates. Finally, pads were connected to the pins using gold wires via the electro-soldering technique. Figure 3b–d [120] shows the optical images of the nanowires. It is seen that the thermal oxidation process assisted in the patterning of the nanowires, with the help of simple shadow masking.

The density and dimension of the nanowires were strongly dependent on the technique used to fabricate them. Figure 3e–g show Scanning Electronic Microscopic (SEM) images of the nanowires. The average ranges of the diameters for tungsten, copper and tin were 20–30 nm, 70–100 nm and 100–250 nm, respectively. The sensors operated on impedimetric sensing, where the changes in the electrical conductance were studied with respect to their interaction with the target analyte at different concentrations. The experimental setup consisted of a flow-through technique where the metallic nanowire-based sensors were deployed on a homemade stainless steel test chamber. The resistance of these conductometric sensors modulated as a result of adsorption or interaction of the chemical species on their sensing surfaces. The lifetime of these sensors is said to be over a year, showing <20% drift in the signal. The detection limits for the three types of nanowires are given in Table 5 [120]. It is seen from the table that the sensors were able to detect the four compounds at very low concentrations.

Another area in which many researchers have worked on food inspection was glucose sensing [121,122,123,124]. Solanki et al. [125] showed the development of impedimetric biosensors for the detection of esterified cholesterol. These sensors have been developed by forming a nanocomposite with MWCNTs and sol-gel-derived silica and chitosan. This nanocomposite was deposited into an indium-tin-oxide glass to form biosensors. Different concentrations of cholesterol were tested to obtain a linearity range and response time of 10–500 mg/dL and 10 s, respectively. The characterization was done using a scanning electron microscope and Fourier transform infrared spectroscopy. The sensitivity and shelf-life of these sensors were 3.8 µA/mM and 10 weeks, respectively. 

One of the works focused on monitoring the quality of chocolate products was shown by Ľubomir Švorca [126]. Impedimetric sensors were fabricated with boron-doped diamond electrodes to determine different concentrations of theobromine (TB). The experiments achieved sub-micromolar LODs using optimized Square-wave voltammetry (SWV) and delivery point validation techniques. The attributes of these sensors are simplicity, fastness, and reliable quantification of the particular analyte. The measurements were done using common voltammetry techniques, such as CV, DPV and SWV in order to evaluate the analytical performances of the sensors under optimized experimental conditions. Three-electrode system was employed, where miniaturized thick-film boron-doped diamond and ceramic substrate were used as working electrode and electrolyte, respectively. Ag/AgCl/3 M KCl was used as reference electrodes, beside screen-printed carbon auxiliary electrodes and silver pseudo electrodes. The pH values of the solutions were tested using a combined system consisted of a pH 1100 L meter and a reference glass electrode. The tested concentrations of TB were ranging between 0.99 and 54.5 µM. The sensitivity of the sensors was 0.07 µA/µM, whereas LODs and relative standard deviation were from 0.42 to 0.51 µM, and from 2.7% to 5%, respectively. 

Parra et al. [127] displayed the fabrication and implementation of sensors from bisphthalocyanine-based carbon paste electrodes for the discrimination of red wines. Three types of rare compounds, including letutium, gadolinium and praseodymium bisphthalocyaninates were used to chemically modify the sensing prototypes. The sensor arrays were used to differentiate six types of Spanish red wines that were made from a variety of grapes, but three different geographical regions and aging stages. The experiments were conducted inside a thermostatised cell where the temperature was kept constant at 20 °C using a liquid system. CV and SWV were used for the measurement purpose, where the carbon-based electrodes, platinum wire and Ag/AgCl/KCl were used as working, counter and reference electrodes, respectively. The measurements were taken at an average of 3–5 times in an aqueous solution of 0.1 M KCl, in order to obtain a stable voltammetric response. The input voltage, frequency of the SWV applied to the electrochemical cell were 100 mV and 15 Hz, respectively. PCA was further applied to the obtained signals, where a division of each wine sample was done to obtain seven of its replicates. 

**Figure 4:**
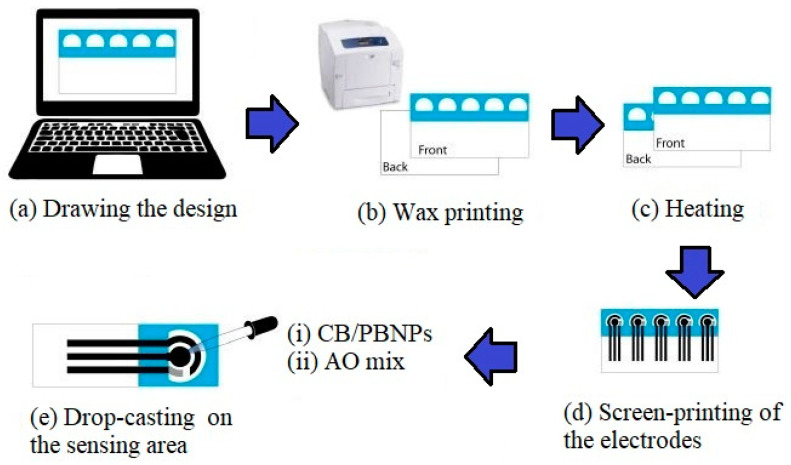
Schematic diagram of the fabrication steps of the paper-based nanomodified sensors [128].

Another work with nanomaterial-based prototypes was done by Cinti et al. [128] to showcase the use of paper-based nano-modified sensors. The sensors were fabricated using a screen-printing technique on common office paper. The processed materials included carbon black and Prussian blue nanoparticles for the quantification of ethanol in beer samples. Figure 4 [128] shows the schematic diagram of the experimental setup for the fabrication process. A total of 100 µL was required to test the amount of hydrogen peroxide in the chosen analyte. CV and EIS techniques were used to test the samples, followed by comparing their results with standardized techniques. Low cost and simple disposal by incineration were two of the major advantages of these sensors. These paper substrates depicted high suitability for sensor development, while depicting similar properties when compared with polyester. Optimization was done on certain analytical parameters, including pH, enzyme, concentration and working potential of the fabricated biosensors. Four categories of beers, namely pilsner, weiss, lager and alcohol-free, were used for the experiments. The sensitivity and LOD of these sensors were 9.13 µA/mM cm^2^ and 0.52 mM, respectively, for the quantified amount up to 10 mM. 

Similar paper-based work was done by Cinti et al. [129] to show the development of three-electrode-based printed sensors. Screen printing and drop-casting techniques were used to fabricate the sensors. The attributes contributed to by the substrates of the sensors are affordability, lightness, portability and biodegradability. An impedimetric sensing process was involved with these sensors, where quantitative measurements were done via studying the change in the range of currents in microamperes levels. The prototypes involved two separate platforms for analysis purposes. The first one was the phosphate part where the voltammetric analysis was carried out. The second one was the nerve agent biosensor, where the amperometric analysis was carried out. Graphite was used to form the working and counter electrodes, whereas silver/silver chloride was used to form the reference electrodes. Similar to the previous work, the performance and specificity of these sensors were improved by carbon black and Prussian blue. Three types of papers were used to form substrates of the sensors. 

Mishra et al. [130] showed the design, fabrication and implementation of stretchable, wearable sensors for the on-site detection of organo-phosphorous chemical threats. These impedimetric sensors have been used as a wearable point-of-care screening tool by using lab-on-a-glove for enzyme-based sensing purposes. The sensing system operated on a real-time basis with wireless data transmission of the responses to a smartphone device. The prototypes were fabricated using stress-enduring inks to form printed electrodes and long serpentine connections. The gloves showed very high resilience and compliance against tested mechanical deformations. CV was used for determining the changes in the responses of the sensors during the characterization and experimental process. The electrolyte was formed using 2 M of potassium ferricyanide in 1 M of KCl. The range of input voltage was fixed between −0.6 and +0.6 V, along with a scan rate of 0.1 V/s. The sensed data were recorded using SWV, having a frequency, amplitude and potential increment of 10 Hz. 25 mV and 4 mV, respectively. The organophosphorus hydrolase-based part of the index finger was used to detect the response of the organophosphate nerve-agent compounds, which was then collected using the thumb finger. 

One of the interesting smart sensing systems that have been used to monitor the food quality is based on radio frequency identification (RFID) sensors, as shown by Huang et al. [131], where wireless sensors have been developed to determine the pH level in the food. The sensors were fabricated with iridium oxide and silver chloride as the conductive material. The electrodes were developed on polyimide substrates to achieve convenient, long-term, and on-demand wireless in-situ detection. Other attributes of these pH sensors were high sensitivity, stability and reversibility. Ubiquitous monitoring of food quality was done in large quantities through these low-cost sensors. The pH sensing mechanism was based on electrochemical reactions taking place with the assistance of a three-electrode system. Iridium oxide and silver chloride were used as working and reference electrodes, respectively. The electrochemical potential was obtained via achieving a redox equilibrium between two oxidation states of iridium oxide. The sensing device was integrated with a battery-less transducer that had an operating principle similar to that of RFID. The wireless system was conducted via inductive coupling between the reader and tag coil antennas contained with tuning capacitors at a resonant frequency. The sensitivity of the sensors was −49.7 mV/pH, operating on inductive coupling between the reader and transponder circuits. This system was useful for products like meat and fish, where their storage over the course of time can change the pH. Table 6 shows the summary of the vital parameters of nanomaterial-based sensors for the detection of food quality.

Other works on the use of nanomaterials for the inspection of food quality are shown in Table 7 [119]. It can be seen from the table that the utilization of nanomaterials has been done to a great extent for a stretch of analyte found in food products. 

### 2.2. Impedimetric Sensors Based on Electronic Nose and Other Smart Sensing Circuits

Irrespective of the nanomaterial-based sensors deployed for food quality measurement, other types of electrochemical sensors are also popular due to their fast response and high sensitivity towards a specific biomolecule. These sensing systems have been used on an industrial scale for the last two decades. The sections below describe a few of the important works done on electronic noses (e-noses) and other forms of electronic circuits used for food quality inspection. The other forms include LC circuits along with intelligent systems embedded with RFID for wireless data transmission.

#### 2.2.1. Electronic Noses

One of the interesting phenomena in the sector of food testing has been done using e-noses [139,140]. The development of these devices for food quality sensing has seen a rapid rise in the last decade. They provide a rapid and early assessment process by mimicking the operating principle and fundamental building blocks of the mammalian olfactory system. They offer advantages such as high sensitivity, non-invasive measurement techniques, rapid and cost-effective detection techniques, and correlation with the human sensor panel [141,142]. Due to these attributes, many food-related applications have employed e-noses for quality control. These devices operate in two categories, namely the pattern recognition of the target analyte, and the determination of quality in terms of classification, flavor detection and spoilage [143]. The sensors used within the e-noses have a strong commonality with the data processing algorithms that analyze the sensed data. They normally contain an array of sensors, where the prototypes are made up of polymers and metal oxides. Sensor arrays mounted inside the electronic noses operate on the impedimetric sensing. The sensing arrays were positioned on replaceable modules so that they can be easily replaced or modified without changing the platform, in case of any malfunction. The sensors presented cross-sensitivity to some of the target chemical compounds. High measurement frequencies were achieved through the reduction in the dead volumes of the sensor chamber. These devices have been widely used to detect the spoilage of a large variety of food products, such as grains, meat, fish and dairy products. 

**Figure 5:**
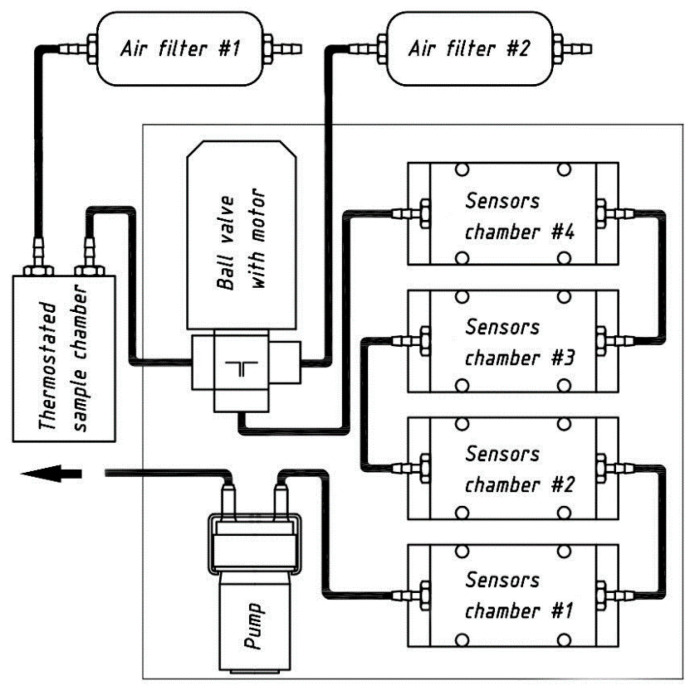
Schematic diagram of the e-nose depicting its different components [144].

Some of the integrated sensing arrays used inside the e-noses include MOSFET, MOX and Taguchi sensors. In this area, Wojnowski et al. [144] explained its use by developing an electronic nose to assess food quality. The nose was used in conjugation with the support vector machine (SVM) method to classify different samples of poultry meat. The prototypes were able to detect the shelf-life of the poultry and adulterations in extra virgin oil with an accuracy of 100% and 82%, respectively. Some of the attributes of the developed prototypes were portability and the ability to obtain solutions to analyze the tested food products. The sensing system consisted of a thermostated sample block, a two-way valve, four replaceable sensor modules that are connected in sequence, two gas filters, and a pump. Figure 5 [144] shows the schematic diagram of the pneumatic assembly of the e-nose. The sensors were connected using a signal-conditioning circuit consisting of a microprocessor, 12 power supply and an analog-to-digital converter. The e-nose consists of sensors that determine temperature, humidity, pressure, and concentration of gases. The sensors that were used in the e-nose were fabricated on FR4 plates using a screen printing technique. Some of the gases measured were carbon monoxide, ethanol, hydrogen sulfide, nitrogen dioxide, sulfur dioxide, ammonia, and other volatile organic compounds. The use of the SVM technique was done to classify the samples of rapeseed oil on the basis of the amount of thermal degradation.

The specific inputs given to the SVM were selected through the interpretations done on the PCA loadings. The training of the SVM algorithm was done using 66% of the randomly chosen data, where 10-fold cross-validation was carried out to obtain the overall classification accuracy. The accuracy was of the SVM algorithm was as high as 98.7%, which could be even 100% via increasing repeatability and shelf-life. The SVM algorithm was also used in conjugation with Radial Basis Function (RBF) kernel to classify the samples of extra virgin olive oil as a function of the volume of the admixed sunflower oil. Statistical analysis was done to determine the features from the ANOVA signals obtained from the six sensors. Around 66% of the data from these signals were chosen at random and used to train the SVM, in order to achieve an accuracy of 82.4% via the cross-validation technique. The limitation of cross-sensitivity and high power consumption, as faced by other sensors, was overcome by the developed e-nose. The experimental process was conducted with a sample and purge time of 20 s and 100 s, respectively.

The aromatic profile of food products such as coffee has also been studied by Servini et al. [145]. The nose was used to study, as an instrumental tool, the sensorial analysis of the coffee filter holders. The experimental process consisted of two holders, each containing one and two cups of coffee, respectively. Espresso coffee samples were used for this process, whose extraction was done in such a way that not many differences were observed in their overall aromatic profiles. The study helped to determine the differences obtained for the pH, titratable acidity, total solids, and caffeine, depending on the types of used filter holders. To monitor the freshness in fish samples, O’Connell et al. [146] demonstrated the use of a portable e-nose. The fish samples taken from the Argentinean lake were introduced inside a chamber consisting of gas sensors. The commercial sensing prototypes were coated with tin dioxide to increase their specificity towards the samples kept inside the chamber. The change in the response of these sensors was recorded to monitor a change in their pattern with time. The analysis of the signals was done using principle component analysis (PCA) technique. 

**Figure 6:**
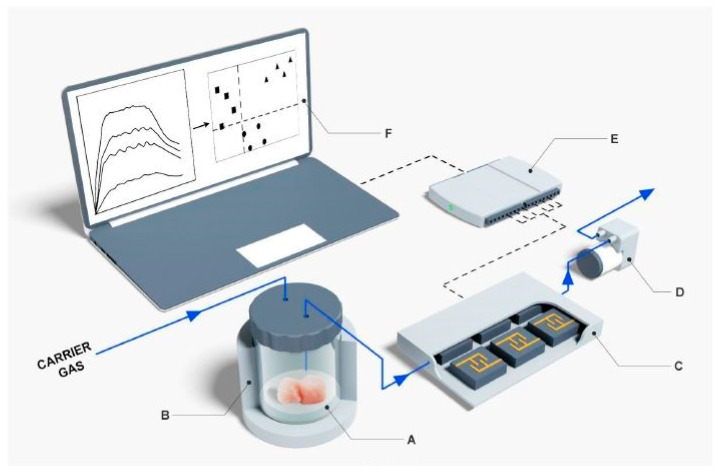
Schematic diagram of the main components of e-nose. (A) Incubation chamber, (B) Sensor chamber (C) Vacuum pump, (D) Analogue-digital converter and (E) Data processing system (F) Scaling done on the data. [147].

The intensity of the signals increased with the increase in the mass of fish as well as the storage time. Some of the samples got rotten with an increase in the number of days of storage, irrespective of the storage conditions. The pattern of the signals changed corresponding to those rotten samples, while the same remained indifferent for the non-rotten samples. Similar to this work, the meat quality was also analyzed by Wojnowski et al. [147] using e-noses. Here, the sensors were formed using volatile compounds that indicated certain parameters of the meat products, such as spoilage and shelf life. These sensors operated on the unique fingerprinting technique based on pattern recognition algorithms. Figure 6 shows the schematic diagram of the main components of the e-nose [147]. 

**Figure 7:**
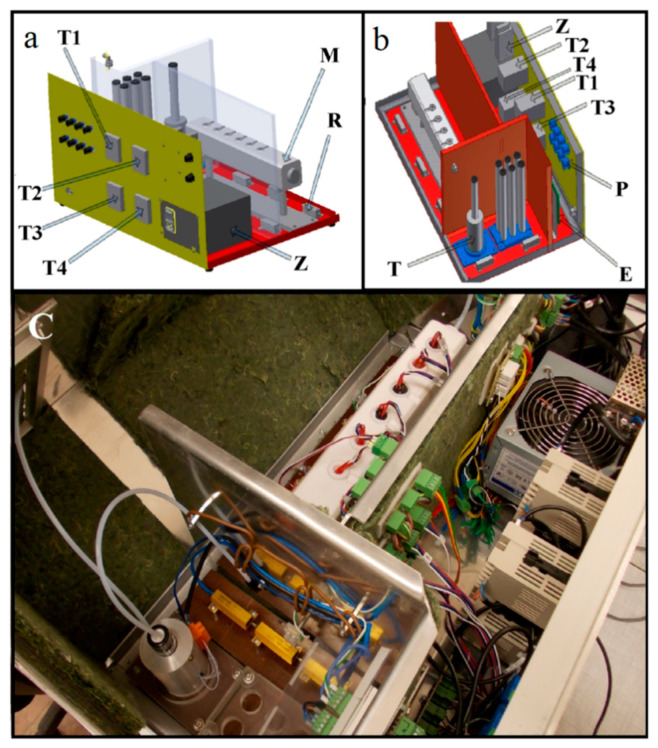
Design of the e-nose used for the classification of five types of honey. (**a**,**b**) Designs of the modules that controlled the temperature of the sensors and sensing chamber present inside the electronic nose. (**c**) Top-view of the entire system. T1, T2, T3 and T4: Modules that control the temperature of particular elements of e-nose, Z: power supply, R: heating element, M: sensors module, P: potentiometer, E: integrated circuit for processing of TGS sensors response signals, T: heating jacket that control the temperature of sample during barbotage [149].

Another work on the categorization of hard cheeses was done by Gursoy et al. [148], where MGD-1 e-noses were used for carrying out the assessment procedure. The ion mobility spectrometric technique was employed to differentiate the hardness of cheese samples based on headspace analysis. Emmental cheese was also studied to determine the changes occurring with age, or due to geographical origin and varieties. The Emmental cheese showed varied responses for samples aged nine months in comparison to the samples aged three and six months. Another food product assessment, as shown by Dymerski et al. [149] with e-noses, was for Polish honey. A few types of honey, such as that sourcing from acacia flower, linden flower, rape, buckwheat and honeydew, were classified using Figaro semiconductor sensors that were embedded inside the e-noses. The process was initiated by providing gradient temperature characteristics using a set of thermostatic modules. The classification process was then carried out using three techniques, namely PCA, linear discriminant analysis (LDA) and cluster analysis (CA). The measurement processes were carried out in optimized conditions by setting up certain parameters, such as volumetric flow rate to 15 lit/h, 35 °C of barbotage temperature, and a sensor signal acquisition time of 60 s. Figure 7 [149] shows the design of the used e-nose. Four different modules were used to control the temperature of the heating jacket, sensors and the sensing chamber of the e-nose.

Adjustments were made to match the relative humidity of the prepared gas and the sample’s temperature. The inert gas flow rate was fixed between 86% and 91%. The entire system was connected to Teflon tubes having a diameter of 4 mm. The sensed data were converted digitally and sent to the computer for analysis purposes using the data acquisition system. The reproducibility and range obtained by the three techniques were 96% and 4.9 to 8.6%, respectively. PCA and CA techniques were able to distinguish three types of honey, whereas LD was able to distinguish all the five types.

Similar to the previous work, an evaluation of agricultural distillates was also done using e-noses, as shown by Dymerski et al. [150]. Six semiconductor FIGARO sensors were used for the experimental process, where the nose was embedded with an electronic circuit for analog to digital conversion of the sensed signal. Optimized measurement conditions were followed with a gas flow rate, thermostat temperature, and sensor signal acquisition time set as 15 lit/h, 15 °C, and 60 s respectively. Three techniques, namely PCA, single-linkage cluster analysis and cluster analysis with spheres, were employed for the interpretation of results. The entire system was connected to Teflon tubes, and a bottle reducer with a metal membrane. This membrane allowed the flow of non-corrosive gases to provide a chemically inert environment for the carrier gases. The recording of the signals was done at specific time instants of 20, 60, 90, 120 and 180 s. The reproducibility and coefficient of variation obtained from the results were 93%, and between 5.8% and 9.2%, respectively. The classification done using PCA showed a response to the change in temperature, where a decrease in temperature caused a corresponding separation of the points associated with high-class agricultural distillates, from that of middle and low-class distillates. Similar changes were observed for the corresponding changes in the volumetric flow rate, where the high-class distillates were away from those of the middle and low class. 

In terms of determining beverages, e-noses have also been used to detect wine where the complexity in each sample, minute disparities present between the wines, and the presence of water and ethanol content had been detected [151]. Certain parameters, such as the assessment of the quality of grapes and its crushing, the fermentation process, the aging of wines in oak barrels, determination of the organoleptic characteristics of the final product, adulteration, and spoilage, are being taken care of by the e-noses. MOSFET and MOX, both of which had been developed with conductive polymers [152], were subsequently used to analyze wines by monitoring the change in the resistance values. Some of the e-noses included surface acoustic wave sensors for precise detection purposes [153]. 

Another work related to the testing of wine was shown by Macías et al. [154]. FIGARO sensors were used to detect aroma and subsequently classify wine samples having an alcohol volume of 12% and 14%. Around sixty prototypes with three different classes of every prototype were collected for the entire operation. The three classes were named alc10, alc12 and alc14. The sensors were embedded in a system alongside an LCD display, two small pumps, and two electro-valves. The cost of the entire system was around USD 200. The system also consisted of temperature and humidity sensors having accuracies of 0.4 °C and ±3.0% RH, respectively. PCA was used for the feature extraction purposes, whereas neural networks (NN) and SVM-based algorithms were used for classification. The accuracies obtained with NN algorithms for both alc10 and alc14 were 100%, and that for alc12 was 99.5%. Other major areas where the usage of the e-noses had been done were microbiological quality control [155] and pharmaceutical industries [156]. Table 8 shows a summary of some of the critical aspects of the work done with respect to e-nose for the detection of food quality. 

#### 2.2.2. Impedimetric Sensors Based on Smart-Sensing Circuits

One of the earlier works related to the development of a sensing system for in situ monitoring of the quality of packaged food was done by Tan et al. [68], where LC sensors were developed with interdigitated electrodes and a spiral inductor. The conducting elements were printed on large substrates, following which their resonant frequency was determined. The application of these prototypes was based on determining the amount of moisture inside packaged cereals. The sensors consisted of three layers: two conducting layers on top and the bottom, and one insulating layer separating them. The printing process was carried out using a toner transfer paper on papers backed with aluminum tapes having a thickness of 10 microns. The printing was completed in three steps. Initially, ferric chloride was used as an enchanting solution, and the printer toner was removed. Finally, the cellulose acetate layer having a thickness of 50 microns was applied to isolate the printed inductor. Thin strips of copper tapes, with a thickness of 20 microns, were finally placed over the insulating layer, in order to connect the ends of the capacitor and the inductor. A resonant frequency between 23–25 MHz was set to perform the experiments. This frequency was varied by varying the dimensions of the spiral inductor and interdigital electrodes. 

The responses were monitored remotely, where the changes in the impedance values were measured for the two detection coils. The resonant frequencies of the sensors were calculated by determining the impedance of the detection coil with an impedance analyzer. The removal of the inductance value of the coil was done when the sensor was absent, in order to determine the values of the response of the sensor. The experiments were conducted by varying the humidity, ranging between 20% RH to 60% RH. The samples were placed inside a testing chamber with increasing humidity to determine the intensity of the spoilage of food content. The resonant frequency changed from 24.35 to 23.8 MHz with respect to the variation of relative humidity from 2% to 44%, respectively. The corresponding responses of the sensors were reversible in nature, having a response time of one and three hours for dry-to-wet and wet-to-dry cycles, respectively. 

A similar work on the use of LC-based sensors for food quality detection was shown by Ong et al. [157], where remote query resonant-circuit sensors were used to determine the growth of bacteria to access the quality control. In-situ detection of three different strains of bacteria, namely *Bacillus subtilis*, *Escherichia coli* JM109, and *Pseudomonas putida*, was done inside a biological medium. The sensed data were transmitted using a loop antenna to detect the complex permittivity of the medium. These sensors have high potential to be commercialized due to their low price and capability of remote query detection. The quality of milk, meat and beer were tested using these sensors. The sensors were placed within the food package in order to obtain the responses of the LC sensors through a loop antenna. The loop antenna had six turns with a total diameter of 9 cm. The solid and liquid testing mediums were treated differently during the experimental process. For the liquid medium, the prototypes were fully immersed, while they were placed on top of a solid medium such that the interdigital electrodes were facing the object. The impedance spectra were studied across the terminals of the loop antenna. The effects of certain factors, such as sensor location, temperature, coating thickness and the absorption of water on the sensing surface, as well as their impact on the responses of the sensors, were also studied.

Some of the impedimetric sensors that have been developed in this domain include aptasensors, [158], which have been developed with several types of conductive materials, depending on the type of food that has been tested.

## 3. Current Challenges and Future Opportunities

Although researchers have been working on the detection of food quality as can be seen above, there still exists some loopholes that need to be worked on in the current scenario. Firstly, most of the sensors described above, especially the ones with graphene and other nanoparticles, have not been embedded with a signal-conditioning circuit. This makes it difficult to deploy these sensors for real-time applications. These sensors can only be used in the laboratory environment, with customized experimental conditions. The impedimetric sensing in actual scenarios would be more complicated due to the intrusion of unwanted particles on the response of the sensors. Moreover, the structural dimensions of the above-mentioned sensors have not been standardized. The researchers have tried to develop prototypes with different processed materials for the sensors, fabrication techniques, and selective material to increase their specificity. This might be encouraging to opt for techniques to develop novel sensors, but the commercialization of these prototypes is not possible. 

Secondly, not much stress on multifunctional sensors has been given yet. The use of multifunctional sensors is very important as it decides the total cost of the system. It also decides the cost of using a sensing system over a prolonged period, since the replacement of a defective sensor every time would neither be cost efficient nor time efficient. Moreover, multifunctional sensors can be employed for different real-time applications in a single scenario. These sensors can also be used as an array of electrodes with selective material for determining the constituents of different kinds of foods and beverages. It would be easier to deploy the multifunctional sensing systems with prototypes being embedded together in a single board in the food-processing industries, rather than using sensors at different places. If these aforementioned points are addressed efficiently, the sensors can be used to a better extent to monitor food quality. 

There are certain issues with e-noses that need to be resolved as well. These instruments lose their sensitivity when they are operated in conditions of humidity. Their sensitivity also decreases when the analyte is present in a high amount. Other issues that the e-noses face are the sensor drift and lack of quantitative data for food products that differ only in smell. These devices need proper calibration before they can be deployed for each application for quality testing. The maintenance of sensitivity can be done by increasing the specificity of the sensors that are embedded within these devices. The array of electrodes used to fabricate the e-noses can be used to increase the versatility of these devices so that each of them can reach out to two or more applications.

Another area where further work is needed is the use of label-free sensors for biosensing applications [159,160]. Although label-based detection has been largely popularized due to certain characteristics, such as the confirmation they provide during bio-molecular sensing, high sensitivity and specificity [161], researchers are nowadays inclined towards label-free detection due to certain reasons. A few of the major advantages of label-free sensors are their simple assessment technique for bio-sensing applications by reducing the liabilities created by the use of labels, the ability to screen the interaction of the chosen cells with specific drugs, quick detection processes due to the avoidance of an additional validation step, the capacity to enable the use of native cells for better biological reference, evaluation of difficult target classes, and the ability to determine the affinity of small molecule inhibitors to particular proteins [162]. Due to the need for long-term operation and stability in the responses, robust techniques of label-free detection have become a necessity. These sensors also provide highly sensitive measurement techniques, due to which the necessity of any kind of tags or specialized reagents is eliminated. Since the researchers are currently working to develop sensors that can provide information about the early-stage diagnosis of a particular anomaly [163], label-free detection would help to determine the real-time kinetic analysis along with quality assessment. The label-free detection would also help to determine a wider diversity of molecules that plays a crucial role in acute and chronic diseases such as cancer, diabetes, obesity, inflammation, neuromuscular disorders, and pathological abnormalities. 

Along with the label-free nature of sensors, the other attributes that should be considered while developing sensors for bio-sensing are the low cost of fabrication, and miniaturization in size. The cost of a single prototype should be as low as possible for roll-to-roll production on an industrial scale, which would be necessary for commercial purposes. Moreover, cheaper sensors would be easier to replace when they are being employed for real-time biomedical and industrial applications. The limited size of the overall dimension of a sensor would help in achieving high sensitivity, low power consumption, additional biomedical uses such as implantable applications, and a long lifetime. The decrease in the size, while maintaining the integrity of the sensors, also helps to reduce the roughness of the surface, which is critical for certain strain-related applications [164,165]. The reduced cost and size of the sensors would carry out the detection process in a non-invasive manner, while the label-free nature would induce an additional level of sensitivity for biological interactions. 

One of the primary goals in the future would be the commercialization of some of the above-mentioned sensing prototypes. This would assist the industries in employing the sensors for testing in real-time applications. There are some commercial sensors [166] that have found success to a certain extent, where the freshness assessment of the different food products has been determined. These prototypes have been categorized as biological sensors having high potential for sensing in smart packing industries. The quality food products have been judged on the basis of their packaging time, before and during the time that is being sold in the stores. One of the cases can be exemplified [167] in the use of sensors containing potassium permanganate (KMnO_4_), where they have been employed as commercial scavengers to reduce the rapid aging of fruits and vegetables. Due to the emission of ethylene from fresh fruits and vegetables, rapid ripening and aging occur in them. The use of these KMnO_4_-based sensors was done for the chemisorption of the overproduced ethylene via the oxidation of ethylene to initially produce ethylene glycol, and subsequently carbon dioxide and water. Another work was shown by Jiang et al. [168], where platinum catalyst on mesoporous silica was used as sensors for removal of ethylene from food products. The sensors were operated at low temperatures, in order to test the samples at a concentration of 50 ppm. Another major example is enzyme-linked immunosorbent assay [169], which has been used for the detection of biomolecules in food products. 

The market for the use of sensors for the detection of food quality has been growing heavily over the last few years [170]. These sensors are mainly sub-classified under biosensors, which are mainly used for the detection of biochemical substances that affect human beings. Some of them are wearable sensors for measuring the physiological parameters of a person [171,172], while some are sensors for smart homes [173,174]. The use of these biosensors has been forecasted to increase from USD 19.2 billion in 2019 to USD 31.5 billion in the next five years, having a compound annual growth rate of 8.3%. This increase is mainly due to the ubiquitous monitoring done on human health to detect acute and chronic diseases such as heart diseases, cancer, obesity, and others. These problems can be avoided to a great extent, with the intake of high-quality food. 

The other innovative forms of sensors that have been popularized are the paper-based sensors, which can be used as degradable and disposable prototypes [175]. These sensors are said to be connected to the internet of things, which allows for the monitoring of the quality of packaged food. The association of these disposable sensors with certain communication protocols, such as RFID and Wi-Fi, makes it easier to determine the response, even from a distance, using a smart device for data collection. The biodegradability of these sensors, due to the use of certain polymers, such as poly (lactic-co-glycolic) acid and polyvinyl alcohol for their fabrication, makes them quite an efficient choice of sensors to be used for healthcare and environmental applications. The change in the dimension of the sensing prototypes from MEMS to smart sensors would make it easier for the detection of the quality of food and beverages to a great extent.

## 4. Conclusions

This paper highlights the research work done on the inspection of food quality using impedimetric sensors. Different types of impedimetric sensors have been displayed, which have been differentiated on the basis of the processed materials used to fabricate them. One of the categories included electrodes formed with nanoparticles such as graphene, platinum and palladium, and other metallic nanowires. These sensors had enhanced the electrical and mechanical characteristics, high aspect ratio, and lightness in weight. The other category included e-noses and other smart sensing systems that have been standardized in the food quality industries due to their fast response and recovery time, high sensitivity and good precision [176]. With the frequency of adulteration in food, which affects the health and quality of life, the need for multifaceted sensors with optimized physio-chemical characteristics has been dire. This makes it a compulsion for the researchers to deploy their sensors for both laboratory and field testing. The specificity and selectivity of the prototypes always seem to be challenged while operating in real-time conditions due to sudden intrusive factors. The monitoring of human health largely depends on equal participation on an industrial as well as on a consumer level, which would assist in the ubiquitous utilization of sensors for food quality tests. 

## Figures and Tables

**Table 1:** Comparative study of the pros and cons of flexible and non-flexible sensors.

Types of Sensors	Pros	Cons
**Non-flexible sensors**	Excellent signal-to-noise ratio Low hysteresis Ability to work in extreme environmental conditions Low power consumption Easy to integrate into systems or modify Small thermal constant	Designing and fabrication include complex procedures High cost due to the requirement of cleanroom facilities All kinds of sensing applications are not possible
**Flexible sensors**	Low-cost Enhanced electromechanical properties Large sensing area Lightweight Higher comfortability High transparency High stretchability High roll-to-roll production	High fragility Needs restricted movement for electrochemical sensing to avoid the effect of flexibility

**Table 2:** Comparative study of selective research work done on the use of carbon-based materials for the fabrication and implementation of sensors for inspection of food quality.

Material	Linear Range	Limit of Detection	Target Product	Reference
Sulphur, nitrogen, mesoporous carbon	0.001–1000 nM	0.00045 nM	Mercury ion	[49]
Multi-Walled Carbon Nanotubes (MWCNTs), Molybdenum disulphide nanosheets (MoS_2_)	0.08–1392 μM	0.015 μM	Chloramphenicol	[50]
Multi-Walled Carbon Nanotubes (MWCNTs), Salen, Cobalt (III)	0.5–6.0 mg L^−1^	0.048 mg L^−1^	Methimazole	[51]
Reduced graphene oxide (RGO)	1 pM–20 pM	1 pM	Kanamycin	[48]
Modified glassy carbon electrode, graphene quantum dots, riboflavin	0.001 μM–1.0 μM	0.2 μM	Persulfate	[52]
Multi-Walled Carbon Nanotubes (MWCNTs)	0.75–20 mg L^−1^	0.5 mg L^−1^	Quinoline yellow	[53]
Molybdenum disulphide (MoS_2_)	300 nM–30 mM	300 nM	Glucose	[54]
Graphene sheet, Nafion, thionine, platinum nanoparticles	0.01–12.0 ng/mL	0.00574 ng/mL	Kanamycin	[55]
Graphene nanosheets, platinum-catalysed hydrogen	0.05–100 ng mL^−1^	0.006 ng mL^−1^	Tetracycline	[56]
Glucose oxidase, aniline, o-phenylenediamine	0.01 to 10 ng mL^−1^	0.007 ng mL^−1^	Streptomycin	[57]

**Table 3:** Comparative study of the research work done on the detection of biomolecules in food using graphene-based sensing prototypes.

Material Used	Analyte	Linear Range	Limit of Detection	Sample Matrix	Reference
Graphene, Polypyrrole, glassy carbon electrode	Dopamine	50–500 nmol/L	7.5 nmol/L	Fish	[105]
Poly (ionic liquids) functionalized polypyrrole, graphene oxide nanosheets	Dopamine	4–18 µM	0.07 µM	Meat	[106]
Reduced Graphene Oxide, gold nanoparticles	Dopamine	10–1000 µM	6 × 10^−2^ µM	Meat	[107]
Polypyrrole, graphene quantum dots	Dopamine	0.005–8 µM	0.00001 µM	Meat	[108]
Graphene sheet, silver hybridized mesoporous ferroferric oxide nanoparticles	Kanamycin	0.050–16 ng/mL	0.15 ng/mL	Pork	[109]
Graphene, Prussian blue-chitosan, nanoporous Gold, Kanamycin antibody	Kanamycin	0.02–14 ng/mL	0.0631 ng/mL	Pork	[110]
Graphene, Nafion, thionines, platinum nanoparticles, anti-kanamycin antibody	Kanamycin	0.01–12 ng/mL	0.0574 ng/mL	Chicken liver	[104]
Copper Indium Sulfide quantum dots, graphene oxide	Kanamycin	0.03–45 nmol/L	0.12 nmol/L	Milk	[111]
Graphene oxide	Clenbuterol	0.001–25 µg/L	15 µg/L	Pork samples	[112]
Poly (3,4-ethylenedioxythiophene), graphene oxide	Clenbuterol	0–250 ng/mL	0.196 ng/mL	Milk	[113]
Perovskite-type barium titanate (BaTiO3) nanoparticles, reduced graphene oxide sheets	Ractopamine	0.01–527.19 µM	1.57 nM	Meat	[114]
Iron oxide nanoparticles, graphene oxide	Ractopamine	0.05–100 µM	0.013 µM	Pork samples	[115]

**Table 4:** Summary of the graphene-based sensors used for the detection of food quality.

Materials	Detection Technique	Target Molecule	Detection Range	LOD	Reference
**Graphene nano-hybrids, platinum NPs**	CV, DPV	Oxalic acid	0.1–50 mM	0.1 mM	[83]
**Graphene nanosheets, palladium NPs**	CV, Amperometry	Hydrogen Peroxide	0.0001–1 mM	5 × 10^−4^ mM	[84]
**Graphene sheets, Titanium oxide, glassy carbon electrode**	CV	Sudan I	3.3 × 10^−6^–6.6 × 10^−4^ mM	6 × 10^−5^ mM	[85]
**Graphene quantum dots, gold NPs**	CV	Malachite green	4 × 10^−4^–0.1 mM	1 × 10^−4^ mM	[89]
**Reduced graphene oxide, Polyamide 6, Polypyrrole**	CV, EIS	Malathion	0.5–20 mM	8 × 10^−4^ mM	[99]
**Laser-induced graphene, Polyimide**	EIS	Citric acid, sodium chloride, L-tryptophan, Sucrose, Guanosine monophosphate	1–1000 ppm	1 ppm	[27]

**Table 5:** Detection limits of the three types of nanowires for the four tested compounds [120].

Nanowires	Nitrogen Dioxide (ng/mL)	Ethanol (ng/mL)	Acetone (ng/mL)	Ozone (ng/mL)
Tin oxide	>1000	5000	15,000	40
Tungsten trioxide	100	25,000	15,000	150
Copper oxide	>1000	40,000	50,000	300

**Table 6:** Comparison of the nanoparticle-based sensors used for the detection of food quality.

Materials	Detection Technique	Target Molecule	Detection Range	LOD	Reference
Tin oxide, Copper oxide, Tungsten trioxide	Change in conductance	Nitrogen dioxide, ethanol, oxygen, ozone	Nitrogen dioxide: 0.1–1 ppm, Ethanol: 5–40 ppm, Acetone: 15–50 ppm, Ozone: 0.04–0.3 ppm	Nitrogen dioxide: 0.1 ppm Ethanol: 5 ppm, Acetone: 15 ppm, Ozone: 0.04 ppm	[120]
MWCNTs, ITO, Chitosan, Silica	CV	Cholesterol	100–5000 ppm	100 ppm	[125]
Boron-doped diamond, ceramic	CV, DPV, SWV	Theobromine	0.99–54.5 µM	0.99 µM	[126]
Carbon Paste	CV, SWV	Letutium, gadolinium and praseodymium bisphthalo-cyaninates	15,000 ppm	15,000 ppm	[127]
Carbon Black, Prussian blue NPs	CV	Ethanol in beer samples	0.52–10 mM	0.52 mM	[128]
Carbon black, Prussian blue NPs, Filter paper, Office paper	CV	Phosphate	10–50 mM	10 mM	[129]
Carbon ink	CV, SWV	Organo-phosphorous	200 μM	200 μM	[130]
Iridium oxide, silver chloride	CV	pH levels in food	2–12	2	[131]

**Table 7:** Uses of nanomaterials to form biosensors for sensing the analyte found in food products [119].

Electrode	Nanomaterials	Analyte	Limit of Detection	Reference
Micro-comb	2-aminoethane thiol, gold nanoparticles	Aflatoxin B_1_	0.10 ng/mL	[132]
Indium-tin-oxide	Chitosan, titanium dioxide nanoparticles	Ochratoxin A	10 ng/mL	[133]
Indium-tin-oxide	Chitosan, gold nanoparticles	Cholesterol	-	[134]
Indium-tin-oxide	Chitosan, cerium oxide nanoparticles	Mycotoxin	0.25 ng/dL	[135]
Glassy carbon	Nafion, room temperature ionic liquid, titanium dioxide nanoparticles, gold nanoparticles	Aflatoxin B_1_	-	[136]
Glassy carbon	Bismuth nano-film	E. coli	100 cfu/mL	[137]
Glassy carbon	Gold nanoparticles	Xanthine and hypoxanthine	-	[138]

**Table 8:** Summary of the above-mentioned works on e-nose on the basis of some vital parameters.

Types of Sensors	Target Material	Reproducibility	Reference
Screen-printed FR4 sensors	Rapeseed oil	High (100%)	[144]
α-FOX sensors	Roasted coffee beans	High	[145]
MGD-1 sensor	Emmental cheese	-	[148]
FIGARO sensors	Honey: acacia flower, linden flower, rape, buckwheat, honeydew	High (96%)	[149]
FIGARO sensors	Triticale, corn, wheat, barley, bread rye, diamond rye and golden rye	High (93%)	[150]
TGS Figaro gas sensors	Three types of wines: alc10, alc12, alc14	High	[154]
